# Biomechanical comparison of two squatting protocols in adolescents and young adults with femoracetabular impingement syndrome

**DOI:** 10.3389/fspor.2026.1659289

**Published:** 2026-01-20

**Authors:** James J. McGinley, Alex M. Loewen, Wilshaw Stevens, Henry B. Ellis, Sophia Ulman

**Affiliations:** 1 Center for Excellence in Sports Medicine, Scottish Rite for Children, Frisco, TX, United States; 2 Department of Orthopaedic Surgery, University of Texas Southwestern Medical Center, Dallas, TX, United States

**Keywords:** biomechanics, hip, kinematics, motion capture, youth, functional assessment, rehabilitation

## Abstract

**Introduction:**

Squatting is an important activity of daily living which can be particularly useful in the management of Femoroacetabular Impingement Syndrome (FAIS). However, double-leg squat protocols can vary significantly, potentially impacting biomechanical outcomes. A squat with a “hold” before the ascent phase represents one variation which increases muscular endurance demands and may elucidate such impacts. The purpose of this study was to identify biomechanical differences between a traditional squat (TSquat) and squat with a hold (HSquat) in adolescents and young adults with pre-operative FAIS.

**Methods:**

Participants (*N* = 46; 35 females; 16.5 ± 1.7 years) undergoing primary unilateral hip preservation surgery for FAIS completed two squat types assessed via 3D motion analysis. The TSquat was performed with descent immediately followed by ascent. The HSquat included a three second pause at participants’ lowest comfortable position. Joint range of motion, moments, powers, and squat depth in participants’ pre-operative limb were compared between each technique.

**Results:**

Participants squatted lower in the TSquat than the HSquat (42.6% ± 12.8% vs. 36.0% ± 13.0%, *p* < 0.001). Maximum hip, knee, and ankle flexion were greater in the TSquat (mean difference: 4.2 ± 0.7°, 10.2 ± 1.2°, 1.4 ± 0.1°, respectively; *p* < 0.05). Peak sagittal moment at the hip/knee (mean difference: 0.10 ± 0.05 Nm/kg, 0.09 ± 0.08 Nm/kg, respectively; *p* < 0.001) and peak power generation at the knee (mean difference: 0.40 ± 0.16 W/kg; *p* = 0.001) were also greater in the TSquat.

**Discussion:**

Participants with FAIS squatted to a shallower depth during the HSquat compared to the TSquat and displayed altered biomechanics, accordingly. Given these findings, greater standardization of squatting techniques is warranted as technique variations may affect outcomes, and researchers must consider potential adjustments made by those with FAIS in their natural squat movement.

## 1 Introduction

Femoracetabular impingement syndrome (FAIS) develops from bony deformity at the hip which produces friction between the femur and acetabulum, increasing the risk of cartilage degradation and osteoarthritis if left untreated (1). Symptoms of FAIS tend to appear most severely when the hip is in deep flexion, internal rotation, or adduction. In both clinical management and surgical rehabilitation, assessing pain and limitations of motion due to hip morphology is informative and can be accomplished by functional movement testing (2, 3).

The double-leg squat has been identified as a particularly valuable assessment tool with a high correlation to daily activity (4). Prior investigations of squatting in FAIS have found altered kinematics and kinetics compared to controls, including decreased squat depth (5, 6). Deeper squats may exaggerate FAIS via increased hip flexion and often internal rotation (2, 7). Despite the clinical relevance of a squat for FAIS and many other lower-extremity conditions (8), standardization of the squat technique in terms of depth, foot placement, and velocity is not apparent in the current literature. Clinicians and researchers may instruct subjects to perform common variations such as the deep hold squat to better control the desired movement or more easily isolate the phases of squat. Subjects may also perform squats differently based on previous training or personal preference. Whether by instruction or subject choice, such variations could impact biomechanical outcomes used in clinical decision-making and confound their applicability.

The deep flexion phase of the squat is particularly pertinent for the pathology of FAIS, and even with posterior pelvic tilt for accommodation, this movement increases the likelihood of the femoral neck meeting the acetabular rim (5, 9). Variations in squat technique alter time spent in the vulnerable phase of deep flexion, providing a clinically relevant population in which to study these potential changes. Therefore, it remains important to identify aspects of the squatting technique which significantly change biomechanical outcomes when not standardized. A squat with a “hold” at the deepest point incorporates a component of muscular endurance and may elucidate additional deficiencies. The purpose of this study was to evaluate the biomechanical differences between two different squatting protocols in participants with FAIS: a traditional double-leg squat (TSquat) compared to a hold squat (HSquat). We hypothesized that participants would adopt a different biomechanical strategy in order to compensate for the increased endurance demand necessary to hold a deep squat position.

## 2 Materials and methods

### 2.1 Participants

Patients undergoing unilateral hip arthroscopy for FAIS hip preservation at a single sports medicine center were invited to participate in a prospective institutional review board approved hip registry (Table 1). Inclusion criteria were participants with a clinical and radiographic diagnosis of FAIS with either cam or pincer morphology. Patients with prior surgical treatment on the hip of interest, unrecovered contralateral surgery, or a secondary syndrome/diagnosis such as cerebral palsy, Charcot-Marie-Tooth, or Down syndrome were excluded. All participants were analyzed prior to ipsilateral surgery. Participants with bilateral symptomatic FAIS and/or prior contralateral surgery were included, but only one limb was assessed per participant. Informed consent was obtained for each adult participant while children provided assent with parental consent. The rights of participants were protected.

**Table 1:** Demographics and Pre-operative patient reported outcomes for patients with femoroacetabular impingement.

Sex	Female: 35 (76.1%); Male: 11 (23.9%)
Age (years)	16.5 ± 1.7 (range 13.5–24.0)
Height (centimeters)	166.4 ± 10.8
Weight (kilograms)	69.5 ± 22.3
HHS (0–100)	63.3 ± 12.6
UCLA activity score (1–10)	6.8 ± 2.8

Mean ± standard deviation. HHS, Harris hip score.

### 2.2 Procedures

Two squat types were employed in this study: a TSquat (10) and an HSquat. Each squat type was performed for three official repetitions after a practice period, with the start of each repetition beginning after the research staff's audible prompt. Participants were instructed to stand comfortably with their feet shoulder-width apart and their arms out in front to ensure motion marker visibility. However, participants were given no instruction with respect to trunk position, foot placement (whether flat or heel raised), squat depth, or the velocity of squat descent and/or ascent to assess natural mechanics. The HSquat was performed first, and participants were instructed to squat down to their lowest comfortable squat depth, hold their lowest position for a count of three, and then return to a standing position. For the TSquat, participants were instructed to squat down to their lowest comfortable point then immediately return to their original standing position (11). After each squat type, participants were asked to state their pain level between 0 and 10, where 0 was defined as “no pain” while 10 was defined as “the worst pain imaginable” (12). To ensure that no participant was pressured to continue with unintended discomfort, participants were continually monitored for signs of visible discomfort or reported pain. A pain score of ≥7 prompted a pause in testing, and the research team confirmed that they could continue the protocol.

During the squat task, participants underwent 3D motion analysis and were instrumented with the Plug-in-Gait (PIG) marker set (13). Additional tracking markers were added on the medial knees, medial ankles, and the most prominent superior region of the left and right iliac crests to account for potential visual loss during the squat. Maximum squat depth was calculated by dividing the vertical displacement of the hip joint center by the highest vertical position of the hip joint center (static standing position) and was utilized to determine a representative trial of each squat type (HSquat, TSquat). Kinematic variables included peak motion of the trunk, pelvis, hip, knee, and ankle. External joint moments (Newton-meter/kilogram) and powers (Watt/kilogram) at the hip, knee and ankle were also compared between the two squat types as a method to identify potential biomechanical compensation. Kinematic data were collected using a 12-camera VICON motion capture system (Vicon Motion Systems Ltd., Oxford, United Kingdom) sampled at 120 Hz. Ground reaction force data were collected using two AMTI force plates (Advanced Mechanical Technology Inc., Watertown, MA, USA) sampled at 3,000 Hz. A Woltring filtering routine with a predicted mean square error of 10 mm^2^ was applied to the trajectory data (14). Squat events for each trial were automatically identified using a custom MATLAB script run within Vicon Nexus (11). The squat cycle included the following: start of descent, end of descent, start of ascent, and end of ascent. The hold period in the HSquat was not assessed for peak kinematic/kinetic values.

Participants also completed a series of questionnaires at the time of testing. The Harris Hip Score (HHS; 0–100) is commonly used to assess pain and overall function of individuals with hip disorders, with higher values indicating a better score (15). Activity levels were assessed via the UCLA activity score, which is a ten-point scale that describes activities ranging from wholly inactive (score of 1) to regularly participating in impact sports (score of 10; (16). These patient-reported outcomes were included given that pain may impact squat depth or the duration that a participant can hold a flexed position, and activity level may improve muscular control even with symptoms of FAIS.

### 2.3 Statistical analyses

Mean and standard deviations were computed for all biomechanical variables. Statistical analyses were performed using SPSS software (Statistical Package for Social Sciences Version 24; IBM, Armonk, NY, USA). Shapiro–Wilk tests for normality revealed deviations from normal distribution; therefore, non-parametric tests were used. Wilcoxon's signed rank tests were performed comparing the biomechanical variables of the two squat types for each participant. Spearman's rank correlations were also conducted to identify an association between the participant's self-reported HHS/UCLA activity scores and the participant's squat depth for each squat type. To consider time-normalized data over the entire squat, mean waveforms and statistical parametric mapping (17) were additionally employed via MATLAB for pelvic tilt, hip flexion, hip internal rotation, trunk lean, hip flexion moment, and knee flexion moment (MathWorks, Version 2024b, Natick, MA, USA). The alpha level was set to 0.05 for all statistical tests.

## 3 Results

**Figure 1:**
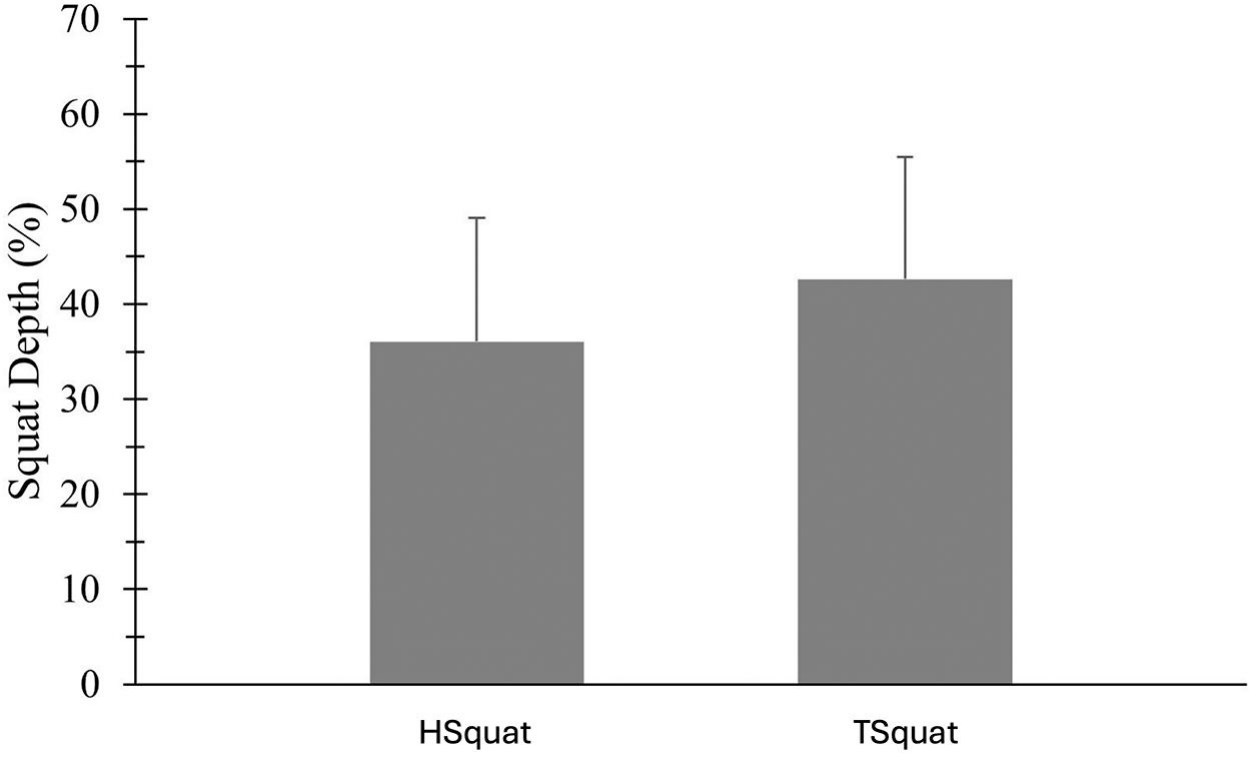
Comparison of squat depth (%) between a hold squat (HSquat) and a traditional squat (TSquat). Subjects with FAIS achieved a deeper squat depth during the TSquat; *p* < 0.001.

All participants (*N* = 46; 35 females; 16.5 ± 1.7 years) were able to complete the squatting protocol. There was no significant correlation between participant's HHS pain or UCLA activity score and maximum squat depth during the HSquat or TSquat (Table 1; see Supplementary Digital Content Figures 1,2, which demonstrates Spearman rank correlations of each squat and patient-reported outcomes). Participants squatted significantly lower in the TSquat (42.6% ± 12.8%) compared to the HSquat (36.0% ± 13.0%; *p* < 0.001; Figure 1).

### 3.1 Comparison of kinematic variables between the HSquat and TSquat

**Figure 2:**
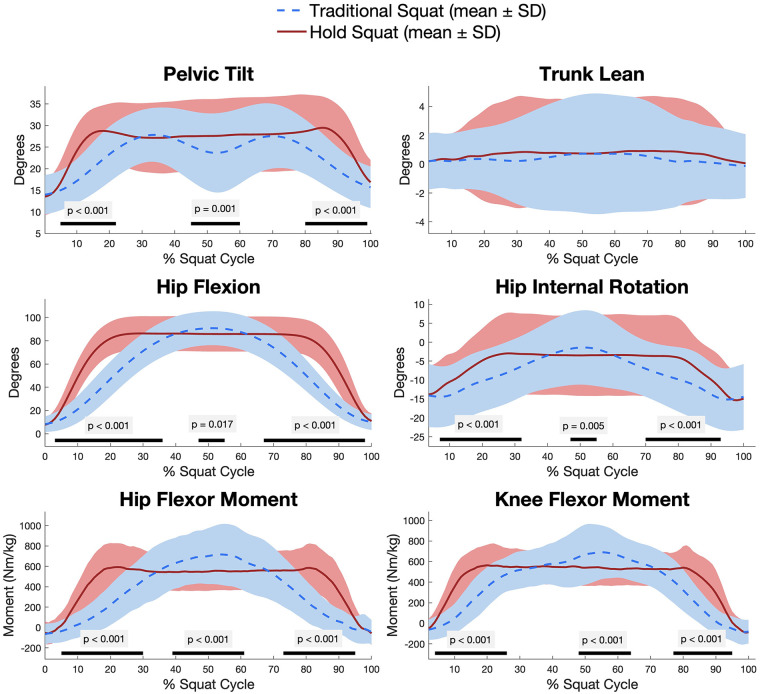
Time-normalized comparison between a hold squat (HSquat) and a traditional squat (TSquat). Lines indicate the mean of the variable of interest, while shading indicates standard deviation (SD). Statistically significant differences in statistical parametric mapping (SPM) are notated by a dark bar below each figure.

Peak kinematic differences between squat types can be found in Table 2. Maximum trunk flexion, trunk lean, and pelvic tilt were not significantly different between squat types. Maximum hip flexion was significantly greater during the TSquat (mean difference: 4.2° ± 0.7°; *p* = 0.002) and participants were less abducted at the hip during the HSquat (mean difference: 1.5° ± 1.7°; *p* = 0.020). There was no significant difference in maximum internal hip rotation between the two squat types. Maximum knee flexion was significantly greater during the TSquat (mean difference: 10.2° ± 1.2°; *p* < 0.001). In addition, there was significantly greater ankle dorsiflexion during the TSquat (mean difference: 1.4° ± 0.1°; *p* = 0.002). Time-normalized statistical parametric mapping between both squat types revealed differences in descent, deep squat, and ascent phases of pelvic tilt, hip flexion, and hip internal rotation. No differences in trunk lean were seen at any phase (Figure 2).

**Table 2:** Maximum kinematic variables between the HSquat and TSquat.

Variable	HSquat	TSquat	*p*-value
Trunk flexion	28.5 ± 12.0	29.8 ± 13.2	0.160
Trunk lean	0.7 ± 3.0	0.4 ± 2.7	0.057
Pelvic tilt	2.7 ± 3.8	2.3 ± 3.4	0.107
Hip flexion	87.7 ± 14.8	91.9 ± 14.1	**0**.**002**
Hip abduction	14.2 ± 6.6	15.7 ± 8.3	**0**.**020**
Hip internal rotation	−0.7 ± 10.8	−0.1 ± 9.7	0.107
Knee flexion	90.2 ± 20.6	100.4 ± 19.4	**<0**.**001**
Ankle dorsiflexion	32.0 ± 6.0	33.4 ± 5.9	**0**.**002**

Mean ± standard deviation. All kinematic values expressed in degrees. Significant differences between the hold squat (HSquat) and traditional squat (TSquat) presented in bold.

### 3.2 Comparison of kinetic variables between the HSquat and TSquat

Peak flexor moments at the hip, knee, and ankle in the sagittal plane can be found in Table 3. Participants generated a greater peak sagittal moment during the TSquat both at the hip (mean difference: 0.10 ± 0.05 Nm/kg; *p* < 0.001) and at the knee (mean difference: 0.09 ± 0.08 Nm/kg; *p* < 0.001). Time-normalized statistical parametric mapping revealed differences in descent, deep squat, and ascent phases for both hip and knee moments, as well (Figure 2). There was no significant difference in peak sagittal ankle moment between the two squat types.

**Table 3:** Maximum sagittal moments between the HSquat and TSquat.

Variable	HSquat	TSquat	*p*-value
Hip flexor moment	0.71 ± 0.24	0.81 ± 0.29	**<0**.**001**
Knee flexor moment	0.70 ± 0.20	0.79 ± 0.28	**<0**.**001**
Ankle dorsiflexion moment	0.41 ± 0.14	0.43 ± 0.16	0.385

Mean ± standard deviation. All moment values are presented in Nm/kg. Significant differences between the hold squat (HSquat) and traditional squat (TSquat) presented in bold.

**Figure 3:**
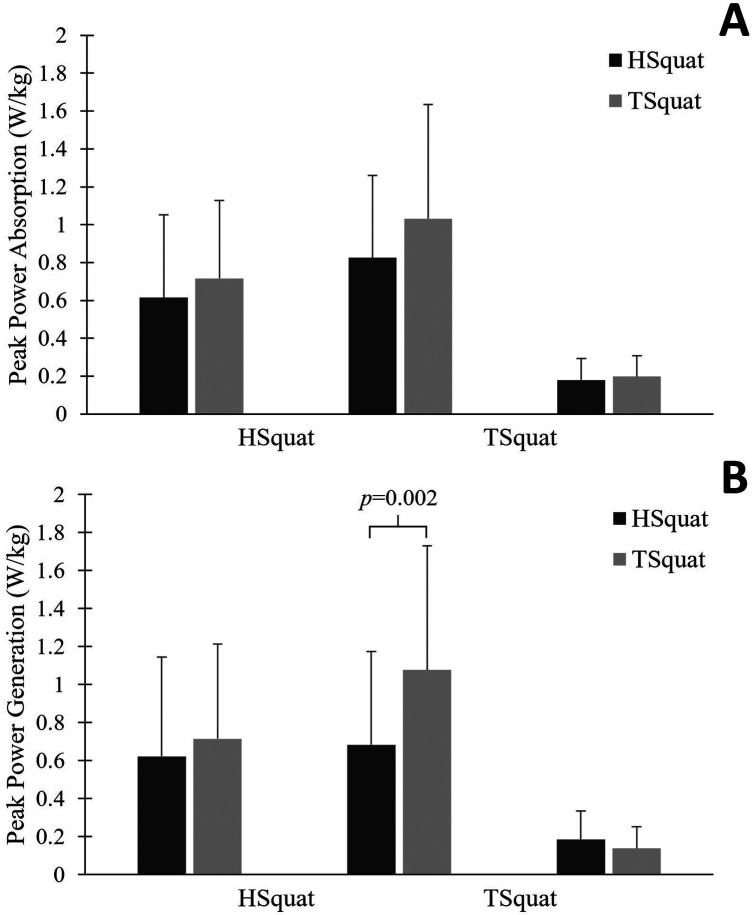
**(A,B)** comparison of peak sagittal power between a hold squat (HSquat) and a traditional squat (TSquat).

There was significantly greater peak power generation at the knee during the TSquat (mean difference: 0.40 ± 0.16 W/kg; *p* = 0.001; Figure 3). No differences in peak power absorption or hip/ankle peak power generation were observed.

## 4 Discussion

The current study evaluated biomechanical strategies adopted by participants with FAIS during a TSquat compared to an HSquat. Participants performed a deeper squat during the TSquat by a difference of 6.6% of their standing height. These results suggest that biomechanical outcomes differ based on the inclusion of a deep hold in a double-leg squat, and trials which do not standardize this technique are susceptible to unintended confounders. One potential explanation for the decreased squat depth in the HSquat is participants’ anticipation of the hold portion. If discomfort is anticipated at the deepest portion of the squat, and the HSquat method forces the participant to hold that for a longer period, participants may compensate by reducing their squat depth. Still, pain was not directly quantified in this study, thus interpretation on avoidant movements remains speculative. Anticipatory alterations in movement were further explored by statistical parametric mapping of the entire squat cycle and mean waveform analysis, identifying changes in movement patterns not only at the deep phase of squat, but also early in descent and late in ascent when normalized by time. These results further corroborate that FAIS participants are capable of maintaining the deep squat during a hold, but they do not reach the same depth as they would without a hold.

Even if anticipation of discomfort impacted the squat, pain measured by HHS did not directly relate to squat depth. Previous work has suggested that the pain associated with the squatting movement is more limiting for range of motion than the bone morphology itself (18). The related concept of kinesiophobia has also been associated to decreased squat depth in adult females with FAIS, though adolescent cohorts have not been explored (19). Therefore, pain or discomfort which limits movement may be more acute while the current study's patient-reported outcomes better indicate long-term function and activity.

Ultimately, the variation in squat depth identified between the TSquat and the HSquat without explicit instructional differences illuminates the need for standardization of the double-leg squat technique. For example, multiple previous studies report kinematic and kinetic variations according to stance width, most notably with increased joint moments at wider widths (20–22). Others identify added counterweights (23) which increase hip dependence or adjusted loading patterns affecting knee extensors (24–26) as ways to alter biomechanics. Of course, squat depth may exaggerate the symptoms of FAIS and is therefore especially beneficial for analysis (2, 3, 7, 27). Without standardization, the current results imply that participant's biomechanics are more dependent on a research center's specific squat type and/or a participant's personal preference than is intended.

Kinematic analysis revealed that maximum hip abduction and flexion at the hip, knee, and ankle were found to be greater in the TSquat than the HSquat. Previous research has affirmed that greater degrees of hip abduction/flexion, knee flexion, and ankle dorsiflexion are required for deeper squats (28–30). Given that squat depth was deeper during the TSquat in this FAIS cohort, greater flexion is to be expected, including a notable disparity of 4.2° in maximum hip flexion. Moreover, almost half of the participants (48%) failed to achieve a hip flexion angle of 90° during the HSquat. A hold squat exercise introduces additional challenges regarding the need for increased neuromuscular control, strength, and the ability to maintain dynamic stability at the lower extremity joints (29, 30). Deep squats with reduced hip flexion may be additionally accommodated by increased lumbar stress, which can have negative impacts (31). In a population such as FAIS who have notable hip pathology and potential biomechanical adjustments, special care may be required to ensure that any squats which include a hold do not incentivize compensatory trunk flexion but do consider a potential decrease in peak kinematic outcomes as a result of the squatting technique.

Finally, kinetic analysis identified that peak sagittal moment at the hip and knee as well as peak power generation at the knee were greater in the TSquat than the HSquat. Again, greater squat depth is likely to lead to a greater joint moment, consistent with prior study (32). Reduced moments can also correspond to less resistance, so a more controlled HSquat could have greater resistive forces and accordingly reduced moments. Though only peak knee power generation was significantly greater in the TSquat, a general trend of greater power generation and absorption was observed. Given that maximum power is usually generated and absorbed at the greatest squat depth, it appears these findings may also stem from biomechanical adjustments associated with the hold. However, power data was not normalized by a time component, so it is possible that participants in the HSquat moved more slowly in late descent or early ascent, which could influence results.

### 4.1 Limitations

Certain limitations must be noted in the current study. Squat order was not randomized, and data was taken from a single representative trial, though all participants were offered practice trials and experienced biomechanists ensured consistent mechanics. While attempting to reduce the impact of fatigue or adaptation, selection bias may be introduced. Bilaterally symptomatic participants were also included, and this may impact double-leg squat movement patterns more than single-leg squats, which are another frequently utilized functional movement test. Future studies should assess other potential variations of the squat such as stance width, foot position, etc. Studies may also investigate a cohort of healthy participants for increased generalizability or consider patient-reported outcomes of pain/kinesiophobia during different squat types. Although statistical parametric mapping offers temporal change insight, future work may include hip joint force using a musculoskeletal model estimate for more precise estimates of impingement beyond the surrogate of external hip joint moment (33–35).

### 4.2 Conclusions

Participants with FAIS tend to squat to a lower depth when performing a traditional squat compared to a hold squat. Potentially as a result, maximum flexion at the hip, knee, and ankle are greater in a traditional squat, as well as peak moments and power generation at certain joints. It is likely that anticipation of the hold alters movement patterns at maximum squat depth. In instances where the kinematic effects of deformity and/or pain are not the primary outcome, hold time should be standardized across a cohort. Regardless of the source of the kinematic and kinetic discrepancies identified in this study, standardization offers greater accuracy in identifying biomechanical abnormalities as well as longitudinal care during rehabilitation. Clinicians and researchers must consider the effect of potential compensatory adjustments in double-leg squat studies of FAIS patients, particularly in comparison to healthy controls better equipped for a deep squat.

## Data Availability

The raw data supporting the conclusions of this article will be made available by the authors, without undue reservation.

## Supplementary material

Supplementary Figure 1 Spearman rank correlations between maximum squat depth (%) and patient reported hip function assessed via the Harris Hip Score (HHS) during a traditional squat (TSquat) and a hold squat (HSquat).

Supplementary Figure 2 Spearman rank correlations between maximum squat depth (%) and subject's overall activity level assessed via the UCLA activity score during a traditional squat (TSquat) and a hold squat (HSquat).
