# Supplementary figures and images for: Biomechanical comparison of two squatting protocols in adolescents and young adults with femoracetabular impingement syndrome
Source: Front Sports Act Living. 2026 Jan 20;8:1659289. doi: 10.3389/fspor.2026.1659289 (PMC12864383; doi:10.3389/fspor.2026.1659289)

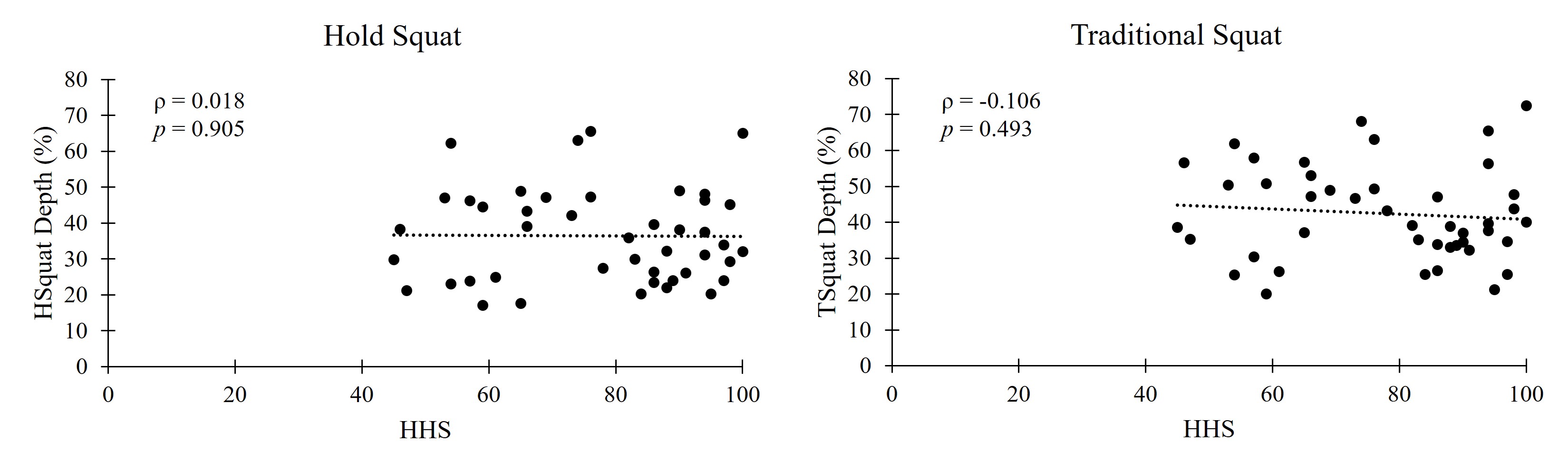

Supplement: Supplementary Figure 1 — Spearman rank correlations between maximum squat depth (%) and patient reported hip function assessed via the Harris Hip Score (HHS) during a traditional squat (TSquat) and a hold squat (HSquat). [file Image1.jpeg]

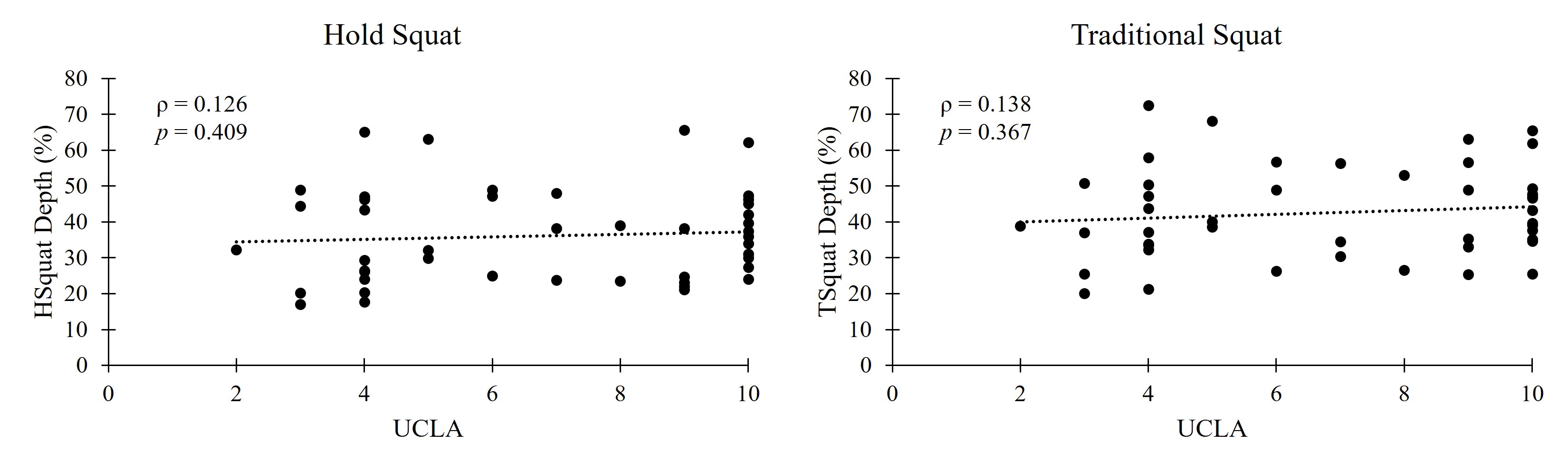

Supplement: Supplementary Figure 2 — Spearman rank correlations between maximum squat depth (%) and subject's overall activity level assessed via the UCLA activity score during a traditional squat (TSquat) and a hold squat (HSquat). [file Image2.jpeg]
